# Short-Term Outcomes of a Healthy Relationship Intervention for the Prevention of Sexual Harassment and Sexual Assault in the US Military: Pilot Pretest-Postest Study

**DOI:** 10.2196/64412

**Published:** 2024-10-29

**Authors:** Belinda Hernandez, Ross Shegog, Christine Markham, Susan Emery, Elizabeth Baumler, Laura Thormaehlen, Rejane Andina Teixeira, Yanneth Rivera, Olive Pertuit, Chelsey Kanipe, Iraina Witherspoon, Janis Doss, Victor Jones, Melissa Peskin

**Affiliations:** 1 Department of Health Promotion and Behavioral Science The University of Texas Health Science Center at Houston School of Public Health San Antonio, TX United States; 2 Department of Population Health Dell Medical School The University of Texas at Austin Austin, TX United States; 3 Department of Psychiatry and Behavioral Sciences University of Texas McGovern Medical School University of Texas Health Science Center at Houston Houston, TX United States; 4 Joint Base McGuire-Dix-Lakehurst Trenton, NJ United States; 5 Workforce Technical Assistance & Training Evaluation Division Defense Workforce Development Center Washington, DC, DC United States

**Keywords:** sexual assault prevention, sexual harassment prevention, healthy relationships, military, technology-based interventions, intervention mapping

## Abstract

**Background:**

Sexual harassment (SH) and sexual assault (SA) are serious public health problems among US service members. Few SH and SA prevention interventions have been developed exclusively for the military. Code of Respect (X-CoRe) is an innovative web-based, multilevel, SA and SH intervention designed exclusively for the active-duty Air Force. The program’s goal is to increase Airmen’s knowledge and skills to build and maintain respectful relationships, ultimately reducing SH and SA and enhancing Airmen’s overall well-being and mission readiness.

**Objective:**

This pilot study aimed to assess the short-term psychosocial impact (eg, knowledge, attitudes, and self-efficacy) of the web-based component of X-CoRe on a sample of junior enlisted and midlevel Airmen.

**Methods:**

Airmen from a military installation located in the Northeastern United States were recruited to complete the 10 web-based modules in X-CoRe (9/15, 60% male; 7/15, 54% aged 30-35 years). Participants were given pretests and posttests to measure short-term psychosocial outcomes associated with SH and SA. Descriptive statistics and paired 2-tailed *t* tests were conducted to assess differences from preintervention to postintervention time points.

**Results:**

After completing X-CoRe, participants had a significantly greater understanding of active consent (*P*=.04), confidence in their healthy relationship skills (*P*=.045), and confidence to intervene as bystanders (*P*=.01). Although not statistically significant (*P*>.05), mean scores in attitudes about SH, couple violence, and cyberbullying; perceptions of sexual misconduct as part of military life; and relationship skills self-efficacy with a romantic partner and friend also improved.

**Conclusions:**

The findings from this study demonstrate X-CoRe’s effectiveness in improving critical determinants of SH and SA, making it a promising intervention for SH and SA prevention. More rigorous research is needed to determine X-CoRe’s impact on SH and SA victimization and the long-term impact on associated psychosocial determinants.

## Introduction

### Sexual Harassment and Sexual Assault in the Military

Sexual harassment (SH) and sexual assault (SA) continue to be serious public health problems among US service members [1]. SH involves unwelcomed sexual advances, requests for sexual favors, and deliberate or repeated offensive comments or gestures of a sexual nature [1]. SA refers to intentional sexual contact characterized by the use of force, threats, intimidation, or abuse of authority; or when the person experiencing SA does not or cannot consent [1]. Compared with rates of SA reported by civilians, those among service members are as high or higher, ranging from 9.5% to 33% for women and 1% to 12% for men [2]. If SH and other forms of sexual violence are considered, the prevalence rates reported by female service members can be as high as 84% [2]. SA is especially prevalent among young service members [3-6]. Approximately 83% to 87% of survivors and 40% to 68% of perpetrators are between the ages of 17 and 24 years [7]. In the military, SA occurs most often between junior enlisted service members who are peers or near peers in rank and are frequently considered friends or acquaintances [8]. Other risk factors for SA in the military include factors across all levels of the socio-ecological model, such as workplace hostility and SH (relationship level) [8], command climate that is receptive to SH in the workplace (installation level) [8,9], and cultural norms (installation level, eg, stigma associated with SH and SA) [2,9,10]. For example, the odds of experiencing SA are 3 and 10 times greater for women and men if they experienced SH in the past year, respectively [8]. Similarly, for women and men who experience workplace hostility, the odds of also experiencing SA double [8].

SA and SH have severe physical and psychological effects on service members, resulting in numerous health problems that impact mission readiness [11], including increased rates of depression [5,12-14], substance use [5,13,14], dissociative disorders [3], anxiety [5,13,14], eating disorders [13,14], sexually transmitted infections [15], suicide and intentional self-harm [3,16], and difficulties readjusting to civilian life after separating from the military [5]. The most common health problem among survivors of SA in the military is posttraumatic stress disorder (PTSD) [3,13]. In one study with a sample of female veterans, women who had experienced SA while in the military were 9 times more likely than those without a history of SA to have symptoms of PTSD [17]. Not only do SA and SH impact individual service members, but they damage the entire military team and degrade unit morale, cohesion, and trust [10,11,18].

### Preventing SH and SA

Although limited, there have been sexual violence prevention programs, many of which are school or university based, that have been developed and scientifically proven to reduce victimization and perpetration [19,20]. These programs primarily focus on preventing perpetration by males [21], victimization among females [22,23], and bystander behavior for males and females [21,24-28]. Despite the proven effectiveness of these approaches, the programs have not been widely adopted, and the prevalence of SH and SA in both military and civilian communities has not substantially decreased [1,29,30]. Additionally, some of these traditional approaches to SA prevention may be perceived as antagonistic to some groups (ie, messages targeting male perpetrators or only females experiencing SA) [30]. Thus, there is a need for more innovative approaches.

Interventions that focus on building healthy relationships are one approach that may be perceived as less antagonistic, reducing the likelihood of negative reactions by certain groups [30]. Not only have these programs demonstrated success in preventing sexual violence in dating relationships, but they also have the potential to reduce other adverse outcomes, such as interpersonal conflicts in the workplace and home, because they build foundational relationship skills (eg, positive communication, anger management, and conflict resolution) [20,30]. To our knowledge, no healthy relationship intervention has been tested for SA prevention in a military setting.

Additionally, a limitation of many current SH and SA prevention interventions is the modality in which they are implemented. Most interventions are delivered in person, with limited opportunities for active engagement or practice [19,31,32], and very few have been developed using technology [19,32]. However, technology can offer several advantages, including increasing fidelity of implementation, the ability to tailor activities by gender or history of sexual violence, greater engagement, and the potential to be disseminated broadly [33-35]. Recent interventions that have used technology for SA prevention have been effective in reducing SA perpetration and increasing bystander prosocial behaviors [21,36-38]. Thus, incorporating technology in SA prevention interventions can be an effective modality.

### Preventing SH and SA in the Military

Despite the problematic SH and SA rates in the military, few SH and SA prevention programs have been developed exclusively for the military [31], and many of the programs implemented lack the principles of effective programming, such as being theoretically based and using well-trained facilitators [39]. To our knowledge, 3 interventions originally developed for civilian college-aged youth, The Men’s Program [40], Bringing in the Bystander [41], and Know Your Power [42], and 2 interventions developed exclusively for military populations, the Navy Sexual Assault Intervention Training Program (SAIT) [43,44] and the Sexual Assault Victim Intervention (SAVI) [45], have been implemented and evaluated at military installations with some success. For example, in a randomized controlled trial of The Men’s Program, men participants reported greater willingness to help, greater bystander efficacy, reduced rape myth acceptance, and reduced intentions of raping and committing SA relative to the comparison group [40]. Pilot studies of Bringing in the Bystander and Know Your Power demonstrated increased bystander behaviors and self-efficacy in acting as a bystander at the follow-up relative to the comparison groups [41,42]. Further, both men and women participants in the SAIT program reported greater rape knowledge and empathy for those experiencing SA, and men participants reported less acceptance of rape myths relative to those in the comparison group [43,44]. Participants in the SAVI program reported improved quality of life, readiness for duty, and coping with sexual trauma compared with those in the control group [45]. Despite their success, there is still a critical need for SA prevention interventions. In a review of the literature on military SA prevention interventions, Orchowski et al [31] identified several gaps, including a lack of leadership engagement within interventions, developmental sequencing of interventions, interventions with sufficient dosage, and interventions that target theoretically and empirically derived risk factors for sexual violence (eg, alcohol use).

### Code of Respect

Code of Respect (X-CoRe) was developed to address the need for SH and SA prevention interventions for the military by the current research team. X-CoRe is an innovative SH and SA intervention that targets multiple levels of the socioecological model (individual, interpersonal, and installation levels) and is designed exclusively for the active-duty Air Force; it is accessible via the web and optimized for mobile phones. The program is accessed through a web-based learning management system (iSpring). Compared with other SA prevention programs, X-CoRe takes a unique approach and focuses on healthy relationships, including peer, intimate, and work relationships. The program aims to increase Airmen’s knowledge and skills to build and maintain respectful relationships, ultimately reducing SH and SA and enhancing Airmen’s overall well-being and mission readiness. The Roman numeral “X” in “X-CoRe” represents the 10 web-based modules that are 10 minutes each and comprise the intervention. The web-based modules consist of a junior enlisted component, who are most at risk for SA [8], and a mid- and senior-level leader component, who are critical in preventing SA [46]. In both components, Airmen learn a life skills or self-regulation decision-making paradigm (select, detect, and protect) that teaches Airmen to select personal boundaries regarding peer, work, and intimate relationships; detect signs or situations that might challenge these boundaries; and use refusal skills and other tactics to protect these boundaries. Modules build on each other and cover topics such as respectful relationships, effective communication in relationships, web-based SH, active consent, alcohol use and consent, reporting incidents of SA, and bystander intervention. Airmen see examples of all types of relationships within the program, including heterosexual and same-gender relationships and situations where males and females are perpetrators and persons experiencing SH and SA. The leadership component also covers using effective communication to respond to disrespectful situations, supporting those who have experienced SH and SA, making referrals to appropriate resources, and preventing SH and SA (eg, creating positive workplace environments and recognizing the role leaders play in preventing SH and SA). A third component of the program is a social marketing campaign designed to target installation norms about relationships. Posters and digital content for social media include messages to foster installation-wide norms that support healthy relationships and reinforce messages received in the web-based component.

A strength of X-CoRe is that it was developed using intervention mapping (IM), a systematic instructional design protocol that integrates behavior change theory, empirical evidence, and input from the community [47]. The development was also informed by extensive input from three Military Advisory Groups consisting of (1) junior enlisted Airmen, (2) midlevel leaders, and (3) subject matter experts (eg, Violence Prevention Integrator, Sexual Assault Response Coordinator, Equal Opportunity, and Family Advocacy). Briefly, following IM's 6-step process, a comprehensive needs assessment was conducted, which included a thorough literature review and in-depth interviews with junior and senior Airmen to identify personal and environmental determinants of SA, as well as best practices for prevention [48]. Matrices of change were then created. These formed the blueprint of X-CoRe and include (1) behavioral objectives (ie, what junior Airmen and leaders are expected to be able to do) as a result of X-CoRe (ie, identify respectful relationships, communicate effectively, and obtain and give consent), (2) relevant determinants of these behaviors (eg, knowledge and self-efficacy), (3) performance objectives for each behavior (eg, decide to have respectful social, intimate, and work relationships and identify and evaluate their own behaviors within past relationships), and (4) change objectives for influencing a change in the determinants of behavior (eg, define respectful relationships and list characteristics of respectful and disrespectful social, intimate, and work relationships). Next, several theory-based methods (eg, knowledge transfer, modeling, and framing) that target these change objectives to influence behavior were identified. Finally, the X-CoRe program was developed with iterative feedback from our advisory groups throughout development.

### This Study

This pilot study aimed to assess the short-term psychosocial impact (eg, knowledge, attitudes, and self-efficacy) of the web-based component of X-CoRe on a sample of junior enlisted and midlevel Airmen. The findings from this pilot study provide critical insight into the potential of an innovative, multilevel intervention to improve psychosocial determinants of SH and SA, ultimately impacting rates of SH and SA in the military. This study fills an important gap in the literature, given the limited number of prevention programs designed exclusively for the military and the few healthy relationship programs designed for SH and SA prevention.

## Methods

### Study Design

We conducted a pre- and postintervention survey among a sample of junior enlisted and midlevel Airmen at a military installation located in the northeast US. Flyers describing the study with the contact information of research staff were distributed via email in February 2024.

### Participants and Study Eligibility Criteria

To be eligible to participate in the study, Airmen had to be on active duty, 18 years or older, and stationed at the local military installation. Airmen of all military ranks were eligible. As shown in Table 1, of the 15 participants, the majority were male (9/15, 60%), White (8/15, 53%), aged 30-35 years (7/15, 54%), ranked as a junior enlisted (E1-E4; 9/15, 60%), and single, never married (7/15, 47%).

**Table 1:** Demographic profile of the study sample.

Demographics		Study sample (n=15), n (%)
**Gender**		
	Female	6 (40)
	Male	9 (60)
**Race**		
	White	8 (53)
	Black	5 (33)
	Hispanic	2 (13)
	Other	
**Age group (years)^a^**		
	19-24	3 (23)
	25-29	1 (8)
	30-35	7 (54)
	36-40	1 (8)
	41-50	1 (8)
**Rank**		
	E1-E4	9 (60)
	E5-E9	6 (40)
	O1-O3	0 (0)
	O4 or above	0 (0)
**Marital status**		
	Married	3 (20)
	Single, never married	7 (47)
	Single/divorced; married/separated	4 (27)
	Cohabitating	0 (0)
	Widowed	1 (7)

^a^Does not equal the total sample size due to missing data.

### Procedures

Airmen who agreed to participate completed an electronic preintervention survey immediately before receiving the X-CoRe program and a postintervention survey immediately after. Airmen completed surveys and all 10 modules on the same day in a private conference room using their mobile phones. Mobile hotspots with Wi-Fi and headphones were provided to ensure the program was accessible and to maintain privacy and confidentiality for participants. Participants were divided into 2 groups based on rank, with junior enlisted in one group and midlevel leaders in another. Two research team members were present in both groups to help troubleshoot technical issues and answer the Airmen’s questions about the program. Each group took approximately 1.5 total hours to complete the survey and program.

### Ethical Considerations

The University of Texas Health Science Center at Houston Institutional Review Board reviewed and approved all study procedures (HSC-SPH-20-0214). Before the pilot study began, participants were given an informed consent form explaining the study procedures, their right to refuse to answer questions, and their right to withdraw from the study at any time. Participants were also informed that they could take breaks at any time if they felt uncomfortable and begin again when they felt ready. Research staff obtained informed consent from all participants after they reviewed the consent form and asked any questions. Participants’ data were stored on a password-protected server hosted by the university and used by authorized study personnel only. All participants were given a unique study identification number before completing the surveys and program for data management and analysis. The same code was used for both surveys. Thus, no names were collected on surveys or within the program, and survey data were anonymous to program staff. Participants were informed that their responses would be confidential and aggregated for reports, manuscripts, and presentations. Although no adverse events occurred during the study, the installation’s Sexual Response Coordinator was available during the pilot to provide support and assistance to any participant experiencing emotional distress while completing the program. Participants received light refreshments to increase comfort while completing the pilot study but were not otherwise compensated.

### Measures

#### Knowledge of SH and SA

To our knowledge, there is no existing knowledge scale that assesses SH and SA as defined by the Uniform Code of Military Justice (UCMJ); thus, 6 true or false questions were developed for this study to assess Airmen’s general understanding of SH and SA according to the UCMJ (Cronbach α=.25). Example items include “Sexual assault is defined by the UCMJ as ‘Intentional and unwanted sexual touching (or attempts to touch) of another person when that person does not give or is not capable of giving consent’” and “It is legal to share a nude photo of a person without their consent.” For analysis, a mean score of the percent correct was calculated, with higher scores indicating greater knowledge.

#### Attitudes About SH

A total of 17 items, adapted from the Sexual Harassment Attitude Scale [49], were used to measure attitudes toward SH (Cronbach α=.76). Participants were asked about their level of agreement with statements reflecting attitudes about SH. Response options were on a 5-point Likert scale ranging from “strongly agree” (1) to “strongly disagree” (4). Example items include “It is normal for Airmen to be sexually teased by others with whom they interact on the job,” and “An attractive Airman has to expect sexual advances and should learn how to handle them.” Two items were reverse coded so that higher mean scores indicate less tolerance for SH.

#### Attitude About Couple Violence

Attitude about couple violence was assessed using the Acceptance of Couple Violence Scale [50] (Cronbach α=0.97). The Acceptance of Couple Violence Scale consisted of 17 items that measured attitudes toward four different types of violence: (1) male-on-female violence, (2) female-on-male violence, (3) same-gender violence, and (4) general violence. Example items include “A man angry enough to hit his female partner must love her very much,” “A woman angry enough to hit her male partner must love him very much,” “A male angry enough to hit his male partner must love him very much,” and “Violence between dating partners can improve the relationship.” Response options were on a 4-point Likert scale ranging from “strongly agree” (1) to “strongly disagree” (4), with higher mean scores indicating less favorable attitudes toward couple violence.

#### Attitude About Cyberbullying

Attitude toward cyberbullying was assessed using the Harmful Cyberbullying Attitudes Scale [51] (Cronbach α=.70). Participants were asked to indicate their level of agreement with 5 statements reflective of attitudes toward cyberbullying. Example items include “Teaching or making fun of others with harmful comments online is fun to me” and “It is alright to send harmful online messages or posts to another.” Response options were on a 5-point Likert scale ranging from “strongly agree” (1) to “strongly disagree” (5), with higher scores indicating less tolerance of cyberbullying.

#### Knowledge of Active Consent

Seven items, adapted from the Revised Sexual Consent Scale [52], were used to measure understanding of active consent (Cronbach α=.72). Example items include “Consent must be given at each step in a sexual encounter” and “Consent for sex one time is consent for future sex.” Response options were on a 5-point Likert scale ranging from “strongly agree” (1) to “strongly disagree” (5). Two items were reverse-coded so that higher scores indicated a greater knowledge of active consent.

#### Perception of Sexual Misconduct as Part of Military Life

Six items, adapted from the Administrator Researcher Campus Climate Collaborative Climate Assessment [53], were used to measure the perception of sexual misconduct as part of military life (Cronbach α=.95). Participants were asked to indicate their level of agreement with statements reflective of SA and SH in the Air Force. Example items include “I don’t think sexual assault is a problem in the Air Force” and “I don’t think there is much that can be done about sexual harassment in the Air Force.” Response options were on a 5-point Likert scale ranging from “strongly agree” (1) to “strongly disagree” (5), with higher mean scores indicating stronger perceptions that sexual misconduct is part of military life.

#### Communication Self-Efficacy in Relationships

Self-efficacy in communicating in relationships was assessed using the Negative Assertion subscale of the Interpersonal Competence Questionnaire [54]. Participants were asked to indicate their level of confidence in handling 8 types of situations with a romantic partner and with a friend (Cronbach α, with a romantic partner is .84; and with a friend is .92). Example items include “Telling a partner you don’t like a certain way he or she has been treating you” and “Saying ‘no’ when a partner asks you to do something you don’t want to do.” Response options were on a 5-point Likert scale ranging from “I am poor at this; I’d feel so uncomfortable and unable to handle this situation, I’d avoid it if possible” (1) to “I’m extremely good at this; I’d feel very comfortable and could hand this situation very well” (5), with higher mean scores indicating greater communication self-efficacy.

#### Healthy Relationship Skills Self-Efficacy

Nine items developed for this study were used to measure self-efficacy for healthy relationships (Cronbach α=.79). Participants were asked to indicate their level of confidence to select, detect, protect, and communicate their personal boundaries within peer, work, and intimate relationships. Example items include “Select personal boundaries within your peer relationships,” “Detect signs and situations that may compromise your personal boundaries,” and “Protect your personal boundaries.” Response options were on a 4-point Likert scale ranging from “not at all confident” (1) to “very confident” (5), with higher scores indicating greater self-efficacy.

#### Bystander Self-Efficacy

Eight items, adapted from the Bystander Efficacy Scale [55], were used to assess bystander self-efficacy (Cronbach α=.90). Participants were asked to rate their level of confidence to perform various bystander actions. Example items include “Express my discomfort if someone makes a joke about another person’s body” and “Get help and resources for a friend who tells me they have been raped.” Response options were on a Likert scale ranging from “Can’t do” (0) to “Very certain” (100), with higher mean scores indicating greater bystander self-efficacy.

#### Demographics

Demographic characteristics collected include gender, race, age, rank, and marital status.

### Analytic Plan

Descriptive characteristics of the study sample were first computed. Paired 2-tailed *t* tests were then conducted to assess differences in psychosocial outcomes from preintervention to postintervention time points, with *P*<.05 indicating statistical significance. Participants’ missing data were dropped from the analysis.

## Results

Table 2 presents the results of the paired 2-tailed *t* tests. As shown, there was a significant increase in knowledge about consent, healthy relationship skills self-efficacy, and bystander self-efficacy from preintervention to postintervention time points. After completing the 10 modules in X-CoRe, participants had a greater understanding of what active consent was (mean difference=.19; *P*=.04), greater confidence in their healthy relationship skills (mean difference=.26; *P*=.045), and greater confidence to intervene as a bystander when witnessing sexual misconduct (mean difference=5.5; *P*=.01). Although not statistically significant (*P*>.05), mean scores in attitudes about SH, couple violence, and cyberbullying, perceptions of sexual misconduct as part of military life, and relationship skills self-efficacy with a romantic partner and friend also improved. Knowledge of SH and SA showed a slight decrease; however, this also was not statistically significant (*P*>.05).

**Table 2:** Change in psychosocial determinants of healthy relationships (n=13).

	Pretest (n=12), mean (SD)	Posttest (n=12), mean (SD)	Mean difference	Paired 2-tailed *t* test (*P* value)
Knowledge of sexual harassment and sexual assault^a^	0.82 (0.11)	0.78 (0.15)	–0.04 (0.19)	.46
Attitudes about sexual harassment^b^	3.9 (0.53)	4.0 (0.53)	0.11 (0.27)	.22
Attitudes about couple violence^c^	3.7 (0.45)	3.8 (0.33)	0.10 (0.24)	.22
Attitudes about cyberbullying^b^	4.6 (0.47)	4.7 (0.45)	0.13 (0.33)	.26
Knowledge about consent^b^	4.6 (0.42)	4.8 (0.36)	0.19 (0.24)	.04
Perceptions of sexual misconduct as part of military life^b^	4.2 (0.50)	4.1 (0.50)	–0.03 (0.10)	.34
Relationship skills self-efficacy: with a romantic partner^c^	3.9 (0.78)	4.1 (0.87)	0.11 (0.78)	.68
Relationship skills self-efficacy: with a friend^c^	3.9 (0.68)	4.0 (0.80)	0.10 (0.58)	.63
Relationships skills self-efficacy: Healthy relationships or Personal boundaries^d^	3.4 (0.41)	3.7 (0.40)	0.26 (0.36)	.045
Bystander self-efficacy^e^	83.6 (20.2)	89.1 (19.3)	5.5 (5.2)	.01

^a^Response options were “Yes” or “No.”

^b^Response options on a 5-point Likert scale of “Strongly agree”, “Agree”, “Neither agree or disagree”, “Disagree”, “Strongly disagree.”

^c^Response options on a 5-point Likert scale of “I’m poor at this”, “I’m only fair at this”, “I’m ok at this”, “I’m good at this”, “I’m extremely good at this.”

^d^Response options on a 4-point Liker scale of “Not at all confident,” “Somewhat confident,” “Confident”, and “Very confident.”

^e^Continuous scale from 0 to 100.

## Discussion

### Principal Findings

This pilot study is among the first to assess the short-term psychosocial impact of an innovative SH and SA prevention intervention for the active-duty Air Force named X-CoRe. We found that Airmen who completed the 10 web-based modules were more likely to express greater knowledge of active consent and confidence in their healthy relationship skills. X-CoRe’s greatest impact was on Airmen’s self-efficacy in intervening as a bystander. Means scores in other determinants also improved, although these findings were not statistically significant. Although a larger, more rigorous clinical trial is needed, the findings from this study demonstrate X-CoRe’s effectiveness in improving critical determinants of SH and SA, making it a promising intervention for SH and SA prevention. Of note, although not statistically significant, knowledge of SH and SA decreased slightly. Post hoc analysis indicated participants’ confusion between the UCMJ definition of SH, specifically regarding hostile work environment and quid pro quo. Future programs should provide further differentiation between these types of SH.

The positive effects of X-CoRe are encouraging, given the influence that knowledge and self-efficacy have on SA perpetration, victimization, and bystander behaviors. Although knowledge of consent alone is insufficient, a positive change in knowledge, along with other theoretical psychosocial determinants (eg, attitudes and intentions), can reduce SA perpetration [21,56]. Similarly, as observed in dating violence prevention interventions and empowerment-based self-defense programs, selecting personal sexual and relationship boundaries and building self-efficacy and skills to protect those boundaries can reduce sexual violence perpetration and victimization [22,57,58]. Additionally, increasing self-efficacy to intervene as a bystander can increase prosocial bystander behaviors, as observed in previous bystander interventions [25,26,59-61].

There are several possible explanations for the positive psychosocial impact of X-CoRe. First, X-CoRe focuses on healthy, respectful relationships, and although the program does not specifically prioritize high-risk subpopulations (eg, lesbian, gay, bisexual, transgender, queer [LGBTQ+]), it is inclusive of all types of relationships, including work, peer, intimate, heterosexual, and same-gender relationships, making the program relatable to many. The program aims to build foundational relationship skills such as effective communication, emotional regulation, refusal skills, and conflict management, core competencies in effective, healthy relationship programs [57,62,63]. Additionally, X-CoRe takes a gender-neutral approach, showcasing both males and females as perpetrators and as persons experiencing SH and SA. Previous SA interventions that have taken a gender-neutral approach have had a significantly greater effect on bystander efficacy compared with those that portrayed those experiencing SA as all or mostly women or perpetrators as all or mostly men [64].

Second, X-CoRe leverages technology to increase participant engagement, tailor activities by rank, and provide opportunities to practice skills through digital role-plays with immediate feedback. These are essential theoretical methods for behavior change [47] and components of effective technology-based health-promoting interventions [33,34]. A recent systematic review of bystander interventions found that only one-third of the 40 programs identified used active learning exercises, skills training, or media (eg, web-based programs or video supplements) [32]. Technology-based interventions for SA prevention, however, are beginning to emerge in the literature and demonstrating effectiveness in reducing sexual violence perpetration [21], increasing prosocial bystander behavior [21,36], and improving psychosocial determinants for SA perpetration and bystander behaviors [21,37,38]. Given the expansion of technology [65] and its educational and implementation advantages [33], more research is needed to develop and test the effectiveness of technology-based interventions for SA prevention.

Finally, a core tenet of the IM protocol is to engage program adopters, implementers, maintainers, and end users throughout the development of an intervention [47]. The benefits of doing so are well documented, including improved health outcomes, behavior self-efficacy, and perceived social support among participants [66-68]. However, decisions regarding military SA prevention or training are often made with little engagement from service members [69]. In this instance, following collaboration principles [70], X-CoRe was developed with extensive iterative feedback from 3 advisory groups comprising junior enlisted Airmen, midlevel leaders, and subject matter experts. The advisory groups provided comprehensive feedback on all intervention components, including the title, look and feel, scenarios, language, characters, and content, increasing X-CoRe’s cultural relevance, motivational appeal, and credibility [47].

### Limitations and Future Directions

Although this study demonstrates the short-term psychosocial impact of X-CoRe, there are limitations to note. First, the sample size was small; however, it was consistent with similar usability studies [37,71-73] and was diverse regarding gender, age, race, rank, and marital status. Still, our ability to detect statistically significant differences among outcomes and assess differences by selected demographics (eg, gender and marital status) was limited due to the small sample size. Second, our study design consisted of a single-group, pre-test-posttest design, which is subject to threats to internal validity and limits our ability to conclusively conclude that our positive outcomes are due to the program. Threats to external validity are also present, given that the study sample consisted of a small sample from a single military installation. Third, our measure for knowledge of SH and SA yielded a low Cronbach α (0.24); however, this may be due to having a small sample size and not necessarily reflective of the measure’s reliability [74]. Studies with larger sample sizes are needed to conduct a full psychometric analysis of the scale and make refinements as needed. Finally, the impact of X-CoRe on long-term psychosocial and behavioral outcomes was not assessed; larger, more rigorous studies are needed.

Future directions of X-CoRe include a randomized controlled trial to determine the impact on SH and SA victimization and long-term psychosocial determinants. If X-CoRe is effective, it can be disseminated more broadly across the Air Force. While X-CoRe was developed for the Air Force, its content and activities apply to other service branches. Thus, plans also include surface-level adaptations to support successful implementation in the other service branches.

### Conclusions

SH and SA are serious public health problems in the US military, and effective prevention interventions are needed. X-CoRe is an innovative, web-based, multilevel intervention designed exclusively for the Air Force. It develops Airmen’s knowledge and skills to build and maintain respectful relationships. Our findings indicate that X-CoRe effectively increases knowledge of active consent, healthy relationship skills self-efficacy, and bystander self-efficacy, making it a promising program for SH and SA prevention. More rigorous research is needed to determine X-CoRe’s impact on SH and SA victimization and the long-term impact on associated psychosocial determinants.

## Abbreviations

IM: intervention mapping
LGBTQ+: lesbian, gay, bisexual, transgender, queer
PTSD: posttraumatic stress disorder
SA: sexual assault
SAIT: Sexual Assault Intervention Training Program
SAVI: Sexual Assault Victim Intervention
SH: sexual harassment
UCMJ: Uniform Code of Military Justice
X-CoRe: Code of Respect

## Data Availability

The data set generated and analyzed during this study is available from the corresponding author upon reasonable request.
